# Secretion of pro-oncogenic AGR2 protein in cancer

**DOI:** 10.1016/j.heliyon.2020.e05000

**Published:** 2020-09-23

**Authors:** Nurshahirah Ashikin Moidu, Nisa Syakila A Rahman, Saiful Effendi Syafruddin, Teck Yew Low, M. Aiman Mohtar

**Affiliations:** UKM Medical Molecular Biology Institute (UMBI), Universiti Kebangsaan Malaysia, Cheras 56000 Kuala Lumpur, Malaysia

**Keywords:** Biochemistry, Cancer research, Cell biology, Enzymology, Molecular biology, Oncology, Protein folding, PDI, Cancer, Secretory pathway, UPR, Protein secretion

## Abstract

Anterior gradient-2 (AGR2) protein mediates the formation, breakage and isomerization of disulphide bonds during protein maturation in the endoplasmic reticulum (ER) and contributes to the homoeostasis of the secretory pathway. AGR2 promotes tumour development and metastasis and its elevated expression is almost completely restricted to malignant tumours. Interestingly, this supposedly ER-resident protein can be localised to other compartments of cancer cells and can also be secreted into the extracellular milieu. There are emerging evidences that describe the gain-of-function activities of the extracellular AGR2, particularly in cancer development. Here, we reviewed studies detailing the expression, pathological and physiological roles associated with AGR2 and compared the duality of localization, intracellular and extracellular, with special emphasis on the later. We also discussed the possible mechanisms of AGR2 secretion as well as deliberating the functional impacts of AGR2 in cancer settings. Last, we deliberate the current therapeutic strategies and posit the potential use AGR2, as a prognosis and diagnosis marker in cancer.

## 1 Introduction

It has been estimated that one-third of the proteome in eukaryotes pass through to the secretory pathway (SP). In this process, nascent proteins are first targeted into the endoplasmic reticulum, the first compartment in the secretory pathway. Here, nascent proteins undergo several maturation steps including protein folding, disulphide bond formation, as well as co- and post-translational modifications. These steps ensure proper structural configurations of proteins before they are released to their destined cellular compartments [1]. Such cellular processes are often tightly guarded and regulated by distinct complementary pathways to maintain ER protein homeostasis (or ER proteostasis) [2]. Dysregulations in any of these steps can disturb the balance of the overall cellular proteome dynamics, leading to diseases [3].

Recently, an important link between ER and tumour development has been established [4, 5, 6]. Notably, the SP has been found to be exposed to a range of environmental pressures in transformed cells. The manifestation of these environmental pressures in the forms of hypoxia, oxidative stress or chemotherapies, aneuploidy, an increase in metabolic demand, and high proliferation rates collectively culminate in tumorigenesis [7]. This can also lead to the accumulation of misfolded proteins in the ER. The ER houses several classical chaperone families, such as the heat shock proteins (HSP), unique chaperones and folding enzymes, and the family of protein disulphide isomerases (PDIs) and peptidyl-prolyl isomerases (PPIases) [8]. Together, these chaperones and folding enzymes are part of the ER quality control (ERQC) system that ensures the proper protein folding. PDI family is one of the major players in protein folding which is categorized by the presence of the thioredoxin motif, CXXC. These enzymes catalyze the formation, breakage and isomerization of disulphide bonds (S–S bonds) of nascent proteins in the lumen of the ER [9].

Within the PDI family, the Anterior GRadient (AGR) family of proteins are one of the key players in the homeostasis of the SP. The AGR family comprises of three members, i.e. TXNDC12 (AGR1), AGR2, and AGR3. In particular, AGR2 protein is the most studied protein in the AGR family that is indispensable for maintaining ER proteostasis [10, 11]. Phylogenetic and bioinformatics studies of the AGR gene family from different species revealed that AGR2 and AGR3 were present in amphibians and higher vertebrates while AGR1 was lost in higher vertebrates [12]. Besides, AGR2 is widely implicated in human diseases, particularly in cancer [12, 13]. There are different *in-vitro* and *in-vivo* models used for dissecting AGR2 functions in health and disease. These models encompass Xenopus, mice, salamander, and mammalian cell lines [13, 14]. Its expression is linked to cellular proliferation as well as the maintenance epithelial tissues in lower vertebrates [12, 15, 16]. In human cancer models, AGR2 was highly expressed across multiple cancer types and its elevated expression is associated also associated with increased cancer cell fitness. For example, it was shown that the AGR2 overexpression exhibited enhanced cancer cell proliferation and metastasis as well as promoting cells survival [17]. Consistent with this, researchers observed an enhanced expression of ER-resident AGR2 in many cancer types [13, 14, 18]. Thus, many studies are clouded around the notion that AGR2 has an intracellular function (iAGR2).

Although AGR2 normally resides within the ER for its protein folding and proteostasis functions, it has been reported that AGR2 can also localize in different cellular compartments such as cytoplasm, plasma membrane and extracellular environment. In recent years, there are accumulating studies that described the presence of AGR2 in the extracellular environment, particularly in the cancer settings. Interestingly, the presence of AGR2 in this extracellular environment demonstrates pro-oncogenic features that has led to a new paradigm on how AGR2 functions. In human, AGR2 proteins are also present in the excretory systems such as blood and urine, suggesting potential clinical utilizations. Despite the detailed characterization of its intracellular functions, the physiological roles of this extracellular AGR2 (eAGR2) remains elusive. Though there have been review articles published discussing the biological functions of AGR2, these were largely focused on the iAGR2 with limited information about its extracellular functions [13, 14, 18, 19, 20]. Hence, this review aims to comprehensively discuss the scientific literatures on the pathological and physiological roles associated with eAGR2 and compare these roles with iAGR2. We will also deliberate on the mechanism of secretion, therapeutic intervention as well as the clinical utility of eAGR2 in the cancer settings.

## 2 AGR2 expression in normal and cancer condition

**Figure 1:**
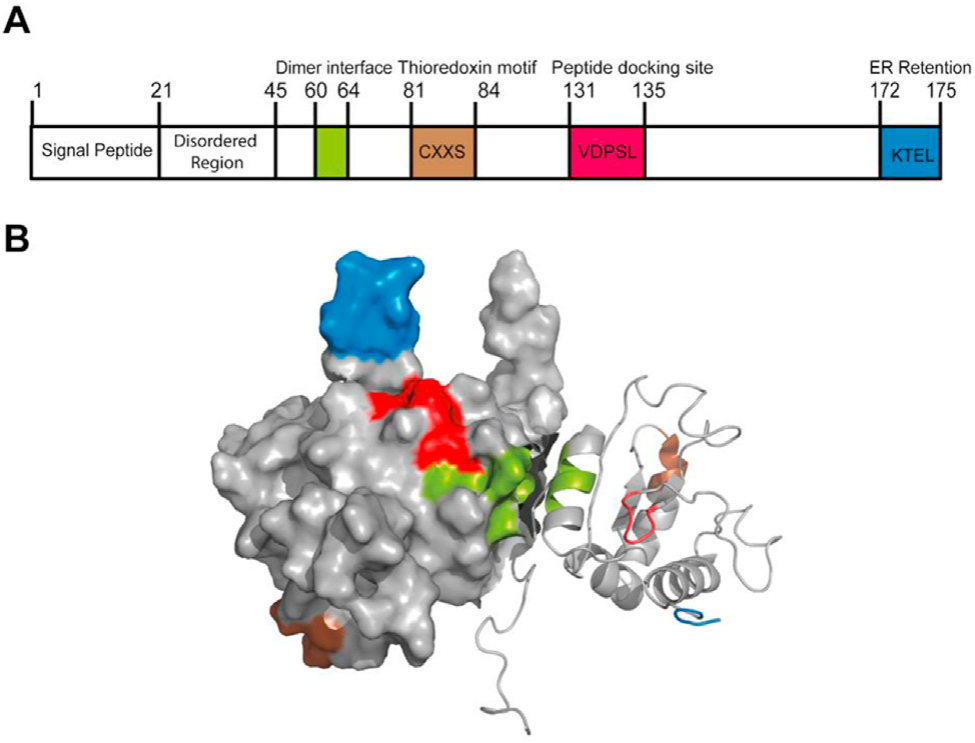
AGR2 protein architecture. (A) Schematic illustrating structure of the full-length AGR2 protein. The panel highlights the functional motifs human AGR2; signal peptide, an N-terminal intrinsically disordered region predicted using PSIPRED v3.3; a dimerization motif (green); a thioredoxin motif (brown), peptide-docking site (red) and ER retention motif (blue). (B) Solution structure of dimeric AGR2 (PDB code:2LNS). One monomer is represented as surface representation and the other monomer represented as a cartoon. The functional motifs in color-coded based on (A).

The human agr2 (hAgr2) gene spans across a 50 kb genomic region of chromosome 7p21.3 [21]. The hAgr2 gene comprises 8 exons and can give rise to seven transcript variants, out of which only four encode protein products (AGR2-203, AGR2-201, AGR2-204, AGR2-202). AGR2-203 is the predominant isoform and hereinafter referred to as AGR2, a ~19kDa proteins comprising 175 amino acids (Figure 1).

Human AGR2 protein is strongly expressed in endoderm-derived organs such as the lung, stomach, colon, prostate and small intestine tissues, and also in organs that contain mucus-secreting cells, or function as endocrine organs [22, 23]. In normal tissues, AGR2 can contribute to the regulation of the total protein loads in the cells. For instance, in the normal mammary gland, AGR2 is upregulated during late pregnancy and lactation period [24]. During this period, high quantity of milk is required, and upregulation of AGR2 assisted by the additional protein-folding and secretory elements are presumably required to promote epithelial cells proliferation and milk protein production to alleviate the increased protein loads. Another line of evidence showed that AGR2 is essential for epithelial barrier. AGR2 is necessary for the production of airway epithelial mucins MUC5AC and MUC5B proteins in respiratory system tissues after allergens encounter [25]. Besides, AGR2 promoter can be regulated by transcription factors FOXA1 and FOXA2 that typically involve in epithelial goblet cells differentiation responsible for secreting mucus in the respiratory, reproductive and gastrointestinal tracts [26].

In human cells, the agr2 gene was first discovered in the estrogen receptor-positive MCF-7 breast cancer cell line [27]. It was later confirmed by both *in-vivo* [28] and *in-vitro* experiments [29] that AGR2 protein expression was indeed regulated by estrogen. AGR2 was also found in other types of cancer that are related to hormone-dependence such as breast cancer [30, 31, 32, 33, 34], prostate cancer [35, 36, 37] and ovarian cancer [38, 39]. Similarly, AGR2 expression was also found in non-hormone dependent cancers such as oesophageal cancer [17, 40], gastric cancer [41], lung cancer [42, 43, 44, 45] and head and neck cancer [46].

Being pro-oncogenic, the overexpression of AGR2 enhanced clonogenic growth and cancer cells survival, rather than inhibiting the growth of the cells in the premalignant Barrett's oesophagus and oesophageal cancers [17]. Loss-of-function assays such as gene knockdown by RNAi showed that AGR2-depleted cells decreased cells proliferation and migration. For example, siRNA-mediated AGR2 knockdown inhibited cells growth, arrested cell-cycle and induced cells death in breast cancer cells [47]. In non-small cell lung cancer, AGR2 promotes cell growth and migration through the AKT signalling pathway whereby depletion of AGR2 reduces the phospho-AKT level [48].

**Figure 2:**
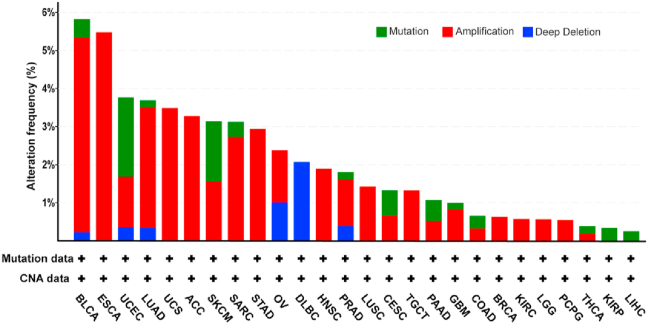
AGR2 genomic alterations in large cancer genomic studies. Genomic alterations in AGR2 was queried using cBioPortal (https://www.cbioportal.org/) that contains 10953 patients in 32 cancer studies [49, 50]. The abbreviations of cancer studies are labelled according to TCGA studies [114].

Large-scale cancer genomics datasets have enabled researchers to look into the genomic alterations of a specific gene [49, 50]. Analysis of The Cancer Genome Atlas (TCGA) PanCancer Atlas studies showed that AGR2 were mainly amplified in the cancer landscape (Figure 2). However, only a few variants, mainly comprising missense mutations (one amino acid change) were reported in these datasets, suggesting that AGR2 mutants have less implication in cancer development. This is consistent with a study showing the lack of association between AGR2 overexpression and genetic or epigenetic changes within the AGR2 gene [51]. Thus, AGR2 is overexpressed predominantly for its oncogenic function. Previous in-silico analysis suggested that there was an absence of CpG island within the 2000 bp upstream of AGR2 promoter region [51]. However, a recent study in colorectal cancer reported that AGR2 can be moderately regulated by DNA methyltransferase 3a (DNMT3a) by directly methylating AGR2 promoter or indirectly via the DNMT3a–SPRY2–miR-194 axis [52]. Events of abnormal AGR2 gene splicing, which are unique in liver cancer, were however expressed at very low levels. Also, small-scale sequencing of all 8 exons of AGR2 gene in five fibrolamellar carcinomas tissues and two human cell lines identified several polymorphisms but no aberrant mutations were found [51]. RNA-Seq was performed on three pairs of lung adenocarcinoma and adjacent normal lung tissues and identified 9 upregulated genes in which AGR2 were among these upregulated genes [43]. Besides, gene expression analysis using the Oncomine database that consists of 7 datasets showed similar upregulation of AGR2, suggesting that AGR2 overexpression supports lung adenocarcinoma pathogenesis [43].

**Figure 3:**
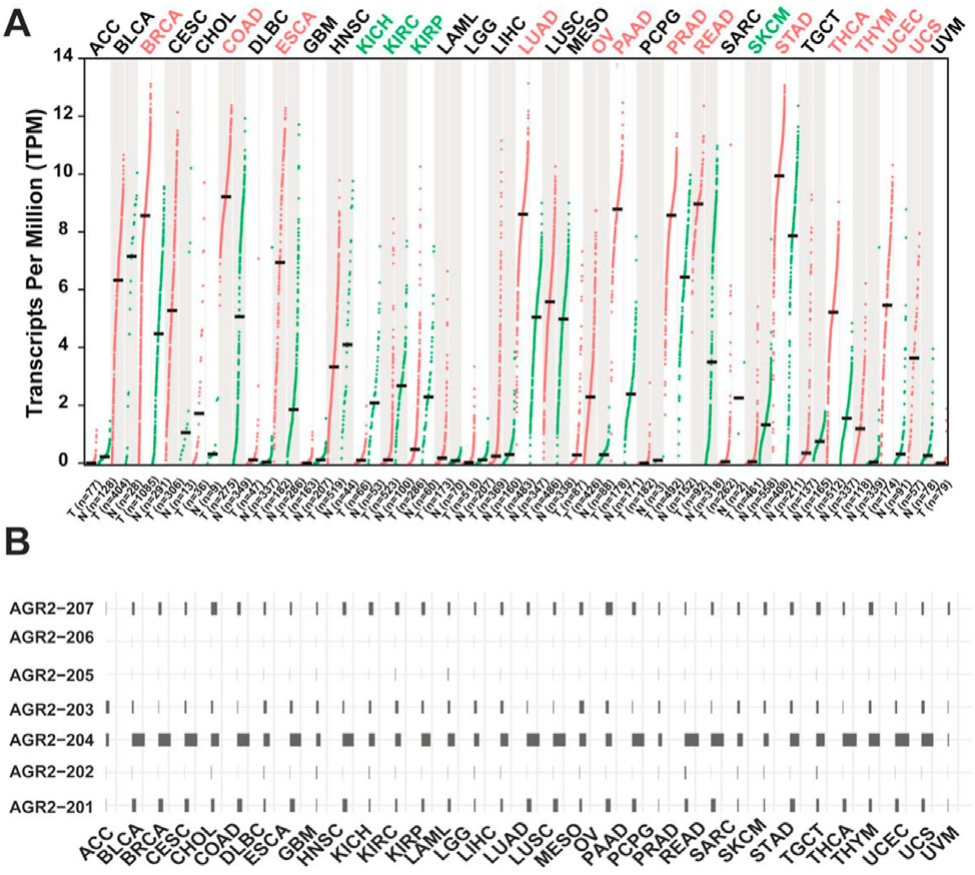
AGR2 and its splice variants mRNA expression in normal and cancer tissues. (A) Dot plots showing AGR2 mRNA expression in tumour and normal tissue samples extracted from the GEPIA2 web tool [115]. Cancer types highlighted in red have significant upregulation of AGR2 expression whereas those highlighted in green have reduced AGR2 expression compared to their normal counterparts. (B) Bar-plot showing the annotated AGR2 isoforms expression across the TCGA Pan-cancer analysis.

Additionally, we compared the AGR2 mRNA expression level using publicly available TCGA and The Genotype-Tissue Expression (GTEx) transcriptomic databases, which curate one of the largest RNA-seq data of cancer and normal tissues respectively. Consistent with studies using human cancer cells and mouse models, AGR2 mRNA expression was significantly upregulated in a broad range of cancer types, such as breast, colorectal, oesophageal, lung, ovarian, pancreatic, rectum, stomach, thymoma, thyroid, and uterine cancers (Figure 3A). However, for kidney and skin cancers, AGR2 was significantly downregulated compared to their normal counterparts (Figure 3A). Basal level of AGR2 expression was observed across different tissue types especially for tissues of epithelial origin. Analysis of AGR2 mRNA isoforms expression using the same datasets demonstrated that AGR2-203 was the isoform predominantly expressed across different cancer types (Figure 3B). While AGR2-201 and AGR2-203 isoforms encode functional proteins and are expressed in cancers, AGR2-202 is weakly expressed across cancers. Interestingly, AGR2-207 that are not annotated to produce any functional proteins are notably expressed in cancers. This suggests possible undiscovered regulatory mechanisms that can modulate the stability and expression of these isoforms, and it is therefore worthy to experimentally determine the expression and roles of these isoforms in cancer.

## 3 Intracellular AGR2

### 3.1 AGR2 as a protein disulphide isomerase

As an ER-resident protein, AGR2 contains a non-canonical ER retrieval motif (C-terminal KTEL) that target AGR2 into the ER (Figure 1) [53]. AGR2 also harbours hydrophobic signal peptide sequences in its first 20 amino acids at N-terminus directing AGR2 import into ER (Figure 1A). Coupled with the degenerative ER retrieval motif and hydrophobic N-terminal signal peptide, it is clear that ER is the AGR2 native resident where it is most functional. Other structural motifs within the AGR2 protein gives a hint about the roles of AGR2. Of note, AGR2 has a thioredoxin fold (CXXS) which taxonomically groups this protein as a member of the PDI family. More than 20 members of the PDI family have been identified in the mammalian ER to date, and AGR2 is the 17^th^ member, hence AGR2 is also called as PDIA17. PDI normally resides in the ER and is responsible for the formation and arrangement of disulphide bonds [54]. Thioredoxin fold is classically described as having a CXXC motif with dual cysteines that is capable in mediating oxidoreductase activity which is important in shuffling disulphide bonds during protein maturation [55]. However, AGR2 only possesses a single cysteine (CXXS) (Figure 1) which may lower its oxidoreductase activity but may, however, contribute to isomerizing the existing disulphide bonds and can also perhaps perform specialised functions in the ER [56, 57]. Despite this, the resolved nuclear magnetic resonance (NMR) structure of AGR2 showed that AGR2 predominantly exists as a homodimer [58]. This dimer is stabilized by Glu60-Lys64 interface in an anti-parallel fashion and the catalytic CXXS motif is held far from this interface (opposing faces). This dimeric structure is therefore suggested to provide equivalent stoichiometric full redox capacity as the canonical thioredoxin motif.

Through its thioredoxin fold, AGR2 apparently enables the folding and trafficking of cysteine-rich proteins that commonly exist in cell surface receptors or membrane proteins that enter the secretory pathway. AGR2 can mediate the maturation of receptors including mucins family such as MUC5 and MUC2 through the formation of mixed disulphide bonds [15, 25]. AGR2 was also required for delivery of EGFR to the plasma membrane that may contribute to oncogenic function in cancers since AGR2-deficient cells showed immature and cytoplasmic staining of EGFR [59]. It was shown that AGR2 can bind to a peptide motif Tx[IL][YF][YF] and this motif is enriched in transmembrane proteins [60]. EpCAM is one of the proteins that harbour the AGR2 biding peptide motif and it has indeed been demonstrated that EpCAM can physically interact with AGR2. Additionally, AGR2 was also required for cell surface delivery of EpCAM presumably through the transient binding at this motif, but it is not known whether the thioredoxin fold has any influence in this interaction. It is likely that AGR2 interacts with EGFR and EpCAM in the ER and facilitates their folding and maturation. AGR2 can also bind to VEGFA whereas AGR2 mutant that lacks the thioredoxin motif failed to bind VEGFA [61]. Collectively, these findings demonstrated that AGR2 catalyzes the proper folding of client proteins via the PDI activity.

### 3.2 AGR2 and ER proteostasis

Intracellular AGR2 overexpression could represent an intermediary entity between the ER and tumour development. For example, AGR2 has been described as a dominant component of ER proteostasis [10, 55]. AGR2 was identified in the proteomics analysis of ER-bound ribosome as a protein that is bound to the newly synthesised cargo proteins [10]. The same study showed that chemically-induced ER stress caused upregulation of AGR2 in liver cancer cells, suggesting that AGR2 is important in ER regulation and quality control. Therefore, an increase in AGR2 expression is perhaps needed to cope with the protein production and secretory demand during cancer development.

ER proteostasis can be perturbed by aberrant intracellular or extracellular signals, including high protein demand, environmental toxins and inflammatory cytokines. Similarly, ER stress - referred to as the accumulation of misfolded or unfolded proteins in the ER lumen, can also impair ER proteostasis. It is demonstrated that under ER stress, cells activate a series of complementary adaptive mechanisms to cope with increased demands for protein folding in the ER. This adaptive mechanism is known as the unfolded protein response (UPR), which is a highly conserved signal transduction pathway [62]. ER stress and UPR activation lead to the development of human diseases such as cancer, diabetes, and neurodegenerative conditions [63]. It was shown that AGR2 expression could be regulated by UPR possibly via IRE1α and ATF6 arms of the UPR and AGR2 silencing disrupts the components of the ER-associated degradation machinery (ERAD), resulting in a reduction in the cells ability to cope with acute ER stress [10]. Related to this, it was also recently shown that proteasome inhibition (MG132 treatment) decreased AGR2 expression and increased UPR markers, IRE1α and ATF4 expression [64]. In pancreatic cancer cell models, AGR2 expression is upregulated upon tunicamycin treatment whereby AGR2 silencing resulted in the overexpression of ER stress marker XBP1s, suggesting a functional role of AGR2 in ER proteostasis [65]. Recently, it was also demonstrated the link between AGR2 dimerization and ER proteostasis [11]. The study showed that under normal or basal condition, AGR2 predominantly exists in homodimer whilst under ER stress, this dimer dissociates in a dose-dependent manner and forms a functional complex with ERAD machinery to sequester misfolded proteins outside the ER. This, in turn, results in the activation of pro-inflammatory signals and AGR2 release into the extracellular environment. This regulation of monomeric-dimeric ratio of AGR2 was shown to be associated with TMED2. This suggests that AGR2 can act as either catalyst for nascent proteins folding or regulator for ERAD, in which both processes are crucial in the stringent requirement of ERQC exit.

## 4 Extracellular AGR2

### 4.1 AGR2 is secreted in cancer

Although AGR2 normally resides within the ER for its protein folding and proteostasis functions, it has been reported that in several cancer types, AGR2 can be found in different cellular compartments such as cytoplasm, plasma membrane and extracellular environment [13, 14]. In pancreatic cancer cells, for instance, AGR2 could escape the ER-retrieval machinery and localize on the surface of the plasma membrane [65]. This provides the first evidence of AGR2 localization on the external surface of cancer cells, rather than solely residing within the ER. It remains poorly understood the roles of this cell surface AGR2, and whether AGR2 can be released into the extracellular environment from this plasma membrane localization.

Secreted proteins that are exported into the extracellular microenvironment represent a major class of proteins, collectively referred to as the secretome [66]. The secretome modulates broad physiological processes, including cellular homeostasis, immune responses, developmental processes, proteostasis and extracellular matrix remodelling. Tumour cells often exhibit an altered composition of secretome compared to the normal tissues. The secretome and the components of the extracellular microenvironment can contribute to the development of cancer [67]. In this context, the presence of AGR2 in the extracellular environment is of particular interest that opens a new paradigm of how AGR2 function in cancer and could possibly modulate the secretome. Here, we summarized all in-vitro studies that described the presence of AGR2 in the extracellular environment of cancer cells (Table 1). AGR2 was found to be secreted in the cultured media of pancreatic, breast, lung, gastric, prostate, colorectal and ovarian cancer cells, implying that overexpression of AGR2 in cancers may be linked to its secretion. In addition, our group identified the presence of eAGR2 expression in the conditioned media from a panel of breast cancer cell lines (unpublished data). It can be speculated that other cancers not listed in Table 1, which have high AGR2 expression, could also secrete AGR2 into the extracellular environment. Taken together, these evidences imply that AGR2 may have essential functions in the extracellular milieu, particularly in cancer emergence.

**Table 1:** Studies describing secreted AGR2 in mammalian cell models.

Cancer	Mammalian cell models	AGR2 implications	References
Pancreatic	BxPC3, MiaPaCa-2, CFPAC-1, HPAC, Panc-1, Aspc-1, SU.86.86	eAGR2 levels correlate with increase rates of pancreatic cancer cell survival, proliferation, and invasion	[116]
	MiaPaca-2	eAGR2 promotes pancreatic cancer metastasis	[77]
Breast	MCF7A, RAMA 37	eAGR2 is O-glycosylated	[93]
	MCF-7, T47D	eAGR2 promotes the IGF-1-induced EMT of estrogen receptor-positive breast cancer cells	[31]
	MCF-7	eAGR2 is detected from cancer-cell spent media. Three times higher than prostate 22Rv1 cells	[117]
Lung Adenocarcinoma	H23, HBEC-AGR2	eAGR2 is secreted in the medium and interacts with ECM components	[74]
Gastric signet-ring carcinoma	Tu-katoIII, HSC-39	eAGR2 activates stromal fibroblasts and stimulates invasion by fibroblasts and cancer cells	[118]
Prostate	PCa, PC3, PC3M-Luc, HUVECs, RWPE-1	eAGR2 directly interacts with VEGFA and promotes metastasis	[61]
	LuCaP	eAGR2 induce the formation of cellular protrusions in prostate stromal cells	[37]
	PC3	eAGR2 promotes prostate cancer metastasis	[77]
	22Rv1	eAGR2 is detected from cancer-cell spent media	[117]
Colorectal	HT-29, SW48	eAGR2 promotes invasion of colorectal cancer cell	[76]
Ovarian	ES-2, A2780, SKOV3	eAGR2 promotes angiogenesis and invasion	[75]
Brain/Glioblastoma	U87, LN18	eAGR2 promotes chemotaxis and tube formation in HUVECs	[71]

### 4.2 Extracellular AGR2 gain-of-function activities and pro-oncogenic features

The secretability of AGR2 in higher vertebrates is a reminiscence of its initial discovery in the lower vertebrate, Xenopus laevis, where XAG-2 was found to be secreted during its development [68]. Furthermore, Kumar et al. showed that nAG, newt AGR2 homolog, was induced during limb generation and the secreted nAG could act as a ligand for salamander-specific cell surface receptor Prod1 [16]. A subsequent study showed that peptide antibody to Prod1 specifically inhibits the proliferation of blastemal (progenitor) cells and S-phase entry activity of secreted nAG, confirming the role of Prod1 as a cell surface receptor for secreted nAG [69].

Emerging evidences have started to shed some lights on the roles of eAGR2 in cancer and tumour microenvironments. The implications of AGR2 in the extracellular environment in mammalian cell models are summarized in Table 1. In pancreatic ductal adenocarcinoma (PDAC) cell line, recombinant AGR2 increased cells proliferation, migration and invasion via signalling pathway mediated by C4.4a cell surface receptor [70]. Consistent with this observation, plates coated with recombinant AGR2 enhances the rate of adhesion of rat mammary epithelial cells, suggesting a role of eAGR2 in cell adhesion [58]. Extracellular AGR2 can also induce the migration and tube formation of human umbilical vein endothelial cells (HUVEC) whereby AGR2 expression is controlled by hypoxia-induced factor-1 (HIF-1), signifying a possible role for extracellular AGR2 in promoting angiogenesis [71]. Li and colleagues showed that eAGR2 formed intermediate interaction with ER-α to induce the expression of insulin-like growth factor-1 receptor (IGF-1), which resulted in enhanced proliferation, migration and EMT process in breast cancer cells [31]. Secreted AGR2 can also be detected in *in-vivo* animal models. For example, AGR2 is secreted and found abundantly in mice colonic mucus and the expression of secreted AGR2 is associated with mucin MUC2 production [72, 73]. Moreover, Fessart et. al described the physiological role of eAGR2 using an organoid model derived from human lung epithelial cells [74]. The study showed that the presence of eAGR2 in the extracellular medium of non-tumoral organoids can convert the non-tumoral organoids to tumour organoid and enhanced their growth about ten-fold. Interestingly, eAGR2 is sufficient by itself to disrupt cell polarisation and promote the acquisition of invasive and metastatic capabilities in which these are independent of its thioredoxin fold and ER-retention motif. Also, both intracellular overexpression of AGR2 and recombinant AGR2 supplementation to the cell culture medium promoted the cell growth of lung cancer [74]. Further, eAGR2 promotes angiogenesis by binding to the pro-angiogenic VEGFA and FGF2 and enhancing their dimerization and activities [75]. Likewise, another study showed that eAGR2 can physically interact with VEGFA through the sole cysteine in its thioredoxin motif, leading to an activation of the VEGFR signalling pathway [61]. The study also showed that eAGR2 significantly promotes the migration and metastatic capability of colorectal cancer cells in the mouse model. Recombinant AGR2 supplementation was shown to upregulate Wnt11 expression that activates the non-canonical Wnt signalling pathway to promote cell migration that consequently suppresses the canonical Wnt/β-catenin signalling pathway [76]. Deletion of the ER-retention motif enhanced the secretion of AGR2 and injecting this purified AGR2 mutant into the prostate and pancreatic cancer xenograft models increased the metastatic capabilities as compared to wild-type AGR2 [77]. The eAGR2 was shown to increase phosphorylation of Hippo signalling component, YAP1 and mTORC2 component, RICTOR (T1135). Moreover, the same study showed that Juxtacrine signalling of eAGR2-mediated juxtacrine signalling enhanced the growth of subcutaneous xenograft prostate cancer cells.

Moreover, eAGR2 has been linked to inflammation. ER stress leads to an increase in AGR2 expression in PDAC development and acquired pro-inflammatory phenotypes [65]. When the conditioned media from ER-stressed PDAC was treated on human normal pancreatic cells, the cells also experienced ER stress and showed a significant upregulation of AGR2. It was also recently demonstrated that the secretion of AGR2 monomers upon ER stress may trigger alarm signals for pro-inflammatory responses in inflammatory bowel disease [11].

## 5 Mechanism of AGR2 secretion

### 5.1 Generation of AGR2 mutants to study its mechanism of secretion

The mechanisms that regulate AGR2 secretion remain elusive despite the well-established pro-metastatic roles of iAGR2. One way to elucidate the functions of the individual motif or domain of AGR2 would be to systematically mutate AGR2. The reported AGR2 mutants have been comprehensively reviewed by Delom et al. [20]. One key motif in AGR2 that is associated with its secretion is the C-terminal ER-retention KTEL, which is crucial for ER localization (Figure 1). The ER-retention motif is classically known as H/KDEL [78]. Proteins containing these motifs prevent ER export and interact with KDEL receptors in the intermediate compartment or the cis-Golgi for ER retrieval. It is understood that variants of the KDEL motif exist [55, 79] to keep proteins inside the ER and this includes KTEL which has a reduced binding affinity for the KDEL receptor [53, 80]. Thus, this non-optimized ER-retention motif KTEL might explain the flexibility of AGR2 to localize in other subcellular compartments, in which AGR2 is capable to ‘leak’ outside the ER and may be essential for dynamic protein function in response to physiological conditions [14, 79, 81].

Mutagenesis of motifs in AGR2 in cell-based systems allowed us to investigate its effects. For instance, deletion of KTEL (ΔKTEL) resulted in AGR2 secretion whereas mutating the KTEL to the optimised canonical KDEL increased the magnitude of AGR2 localisation in the ER [81]. Gupta et al. showed that AGR2 induced the expression of amphiregulin (AREG) and CDX2 only with the wild-type KTEL but not with KDEL [79]. Hence, variants of the ER retention sequence may serve a specific functional role, and in the case of AGR2, this role is served specifically by KTEL. Deletion of this motif eliminates the ability of AGR2 to stimulate cell growth in clonogenic assays and attenuate the p53 transcriptional response to DNA damage [17]. Deletion of the KTEL motif causes AGR2 to be more highly secreted than the wild-type, suggesting that the motif may be processed for AGR2 secretion. Jia et al. demonstrated that the deleted KTEL mutant failed to produce recombinant AGR2, suggesting that the KTEL is important in maintaining the AGR2 structure [61]. Also, a recent study in newt showed that the addition of myc-tag at the nAG C-terminal led to enhanced protein secretion into cell culture media [69]. This is not surprising since the addition of extra myc-tag may hide the KTEL motif and therefore weaken its binding affinity to KDEL receptor. As a result, the protein is not retained inside the cell and instead is secreted into the media. Therefore, in designing plasmid vectors for AGR2 studies, epitope-tag should be fused upstream of the KTEL sequence for more accurate AGR2 localization and functional assays.

Interestingly, eAGR2 is secreted from the ER as a fully functioning protein and it does not merely accumulate as a product of cell death and lysis. A recent study demonstrated that even when the KTEL motif is completely deleted or mutated (i.e KDEL, K172D, K172, AGR2 ΔKTEL), AGR2 can still be secreted at relatively equal levels to wild-type AGR2 although the former has a significantly higher secretion [74]. AGR2-ΔKTEL or AGR2-KDEL mutants, however, did not show any significant difference in eAGR2 secretion levels. This indicates that this motif might not be necessary for AGR2 secretion and AGR2 is likely secreted as a soluble protein. In addition to the KTEL ER-retention signal, the substitution of Cys to Ser at amino acid 81 (Cys81Ser) also results in AGR2 secretion, suggesting that this substitution is important in controlling AGR2 retention in ER [72]. Overall, this implies that AGR2 is dependent on both the KTEL retention signal and the single Cys81 for its ER retention and that mutation on this position to Ser might allow higher levels of AGR2 to be secreted. However, further studies are required to test whether this secretion could be due to AGR2 misfolding upon Cys to Ser substitution.

The NMR structure showed that AGR2_41-175_ can exist in homodimer structure through a novel amino acid motif at position E60-K64 in an antiparallel arrangement of the α1 helix [58]. However, it is worth to note that the protein used in the study is not a full length or mature form since amino acid 1–40 is truncated. AGR2 can also form an alternatively shaped dimeric structure in which oxidation-dependent homo-dimerisation occurs through Cys81-mediated disulphide bond formation which would re-orientate the dimer into a different conformation [82]. Another study supported this observation showing that mutation of Cys81 to Ala prevents peroxide catalyzed dimerization of AGR2, suggesting the sole cysteine is significant for covalent dimer formation [83]. By using an ESI mass spectrometry, Clarke et al. demonstrated that low levels of a chemical oxidant promote an intermolecular disulphide bond through the formation of a labile sulfenic acid intermediate (RSOH). The N-terminal disordered region (amino acids 20–40) is another determinant in AGR2 dimeric structure where deletion of the first 45 amino acids (AGR2 Δ45) significantly stabilises the dimer. A recent study utilized this dimeric/monomeric mutants of AGR2 to investigate whether dimerization has a role in the secretion of AGR2. To this end, Maurel et al. generated monomeric (AGR2 E60A) and dimeric mutant (AGR2 Δ45). The results showed that monomeric mutant AGR2 E60A has a higher secretion rate than the wild-type AGR2 and on the contrary, dimeric mutant AGR2 Δ45 was retained inside the cell, indicating that alteration of AGR2 dimeric versus monomeric status impacts on AGR2 export into the extracellular environment [11].

### 5.2 Pathways for secretion

The common pathway for protein secretion is via the ER and Golgi Apparatus (GA), which is also known as the conventional secretory pathway. A signal sequence is required to facilitate the translocation of the peptide to the ER and to target it to the secretory pathway [84]. Yet, some proteins, which are “leaderless” or lacking a signal sequence can bypass the ER-GA path prior to secretion, by an alternative secretory pathway that is called unconventional protein secretion (UPS) [85]. The presence of highly conserved N-terminal signal peptide (Met1-Ala20) in all AGR2 variants suggests that AGR2 protein processing follow the classical secretory pathway instead of the unconventional one (Figure 1A) [79]. However, it is not known how long does AGR2 stays in the ER or can escape the ER upon ER-signaling cues. It is also unclear whether AGR2 is capable of bypassing ER-GA and follows the UPS. Recently, it was shown that secretion of AGR2 is associated with TMED2, and TMED2 overexpression alters AGR2 dimerization and promotes the secretion of AGR2 monomers possibly through the endo-lysosomal system [11].

ER stress and UPR can be chemically induced by ER-stressing agents such as brefeldin A that blocks ER-GA transport which results in the accumulation of unfolded proteins in the ER. It has been shown that activation of ER stress trough treatment with ER stressors can induce UPS as in the case of mutant CFTR and pendrin [86, 87]. Additionally, ER stress that activates UPR has been shown to increase the secretion rate of ERdJ3 chaperone that is important in extracellular proteostasis activities [88]. These hypotheses might be transferable to other ER chaperones such as AGR2. It is therefore interesting to investigate the possibility of ER stress or UPR activation can cause high secretion of AGR2. There is also a possible interplay between UPR/ERAD/Autophagy as recent studies showed that AGR2 can be secreted through the autophagy-lysosome pathway [11, 64]. Intriguingly, database mining of AGR2 binding peptide motif, TxIYY showed that this motif exists in USP19 [60]. USP19 protein has been linked to UPS by activating a protein quality control mechanism called misfolding-associated protein secretion that sequester misfolded cytosolic proteins [89]. Thus, there is a possibility that AGR2 can function through this USP19 axis and studying the roles of AGR2 in UPS would open new research avenues.

### 5.3 Post-translational modifications associated with AGR2 secretion

#### 5.3.1 Proteolytic cleavage

AGR2 harbours hydrophobic signal peptide sequences in its N-terminus (Met1-Ala20) to direct AGR2 into the ER (Figure 1A). The N-terminal leader sequence targets nascent polypeptides directly from the ribosome to the ER membrane via a signal recognition particle (SRP) and is later cleaved by signal peptidase in the ER lumen, yielding a mature, processed form of a protein [90]. Cell-based analysis of AGR2 with and without signal peptide using immunofluorescence suggested differential subcellular localization whereby the full-length protein is localizing predominantly to the ER and the shorter, mature AGR2 lacking the N-terminal leader sequence localising primarily to the nucleus [81]. As described by Vitale and Denecke, co-translational cleavage by signal peptidase of protein harbouring signal peptide is essential for protein folding and maturation [90]. Thus, protein without a signal peptide would not be directed to the ER lumen, and instead, would demonstrate subcellular mislocalisation. Further, it has also been indicated that signal peptide peptidase can cleave intramembrane signal sequences, leading to the perturbation of ERAD to control the UPR modulators [91]. Studying whether AGR2 involves in proteolysis by signal peptide peptidases and impact upon ERQC, UPR, and disease mechanisms would gain more insights in AGR2 signalling. As discussed previously, current data showed that deletion of KTEL causes the secretion of AGR2 into extracellular media, but whether there are proteases that cleave at this motif is not known.

#### 5.3.2 Disulphide bonds

As described previously, AGR2 sole cysteine residue (Cys81) has been demonstrated to form mixed disulphide bonds with some of its client proteins such as MUC2 that is crucial in mucin productions [15, 72]. This mixed disulphide bond is formed between Cys81 with the cysteine-rich N- and C-terminal domains of MUC2, resulting in the AGR2-MUC2 complex. When Cys81Ser AGR2 mutant was introduced, the interaction between AGR2 and MUC2 was abolished. However, this AGR2 mediated processing of MUC2 is thought to happen intracellularly as AGR2 is found to be localized to the ER in intestinal epithelial cells but not in secretory granules or in the intestinal lumen suggesting that AGR2 does not co-secrete with MUC2. It was also demonstrated that AGR2 homo-dimerizes through an intermolecular disulphide bond formed with Cys81 [82,83]. Ryu et al. for example showed that chemical crosslinking to human colon cancer cells, Hct8 cells, followed by immunoprecipitation of AGR2 and Western blotting identified both monomeric and dimeric AGR2 bands [82]. This dimerization of AGR2 is required for complex formation with BiP/GRP78, an initiator in the UPR pathway and this complex has been shown to attenuate ER stress-induced cell death. Only one study thus far showed that the presence of a single cysteine within the AGR2 thioredoxin-like domain is required for the control of protein secretion [72]. Mutations of Cys81Ser or ΔKTEL caused secretion of AGR2 in CHOK1 cells, where the former showed the highest secretion. It is, therefore, possible that there is an interplay between the KTEL motif and the single Cys in regard to its secretion.

#### 5.3.3 Glycosylation

Most secretory proteins that enter the SP are post-translationally modified by the glycosylation process which is initiated in the ER [92]. Proteins can either become N-glycosylated or O-glycosylated depending on the types of sugar added, though AGR2 seems to be modified by the latter. Clarke and co-workers demonstrated that secreted AGR2 in human and rat mammary epithelial cells is O-glycosylated, with no trace of N-glycosylation detected [93]. This suggests that O-glycosylation, whereby oligosaccharides are linked to the hydroxyl group of serine, threonine, hydroxylysine, or tyrosine might be vital for eAGR2 function. Although the precise nature, number, locality and functions of O-glycan groups that can be added to AGR2 is not fully known, six possible O-glycosylated residue(s) were predicted using the NetOGlyc 4.0 server [94]. Interestingly, all six predicted sites localize within the unstructured N-terminal region, i.e. T23, T24, T33, S36, T43 and T45. This is in agreement with a finding which reported that O-glycosylation sites are located preferentially in unstructured protein regions, in contrast to N-glycosylation [95]. However, in another study that used non-tumour organoids overexpressing AGR2 (HBEC-AGR2), the detected eAGR2 was not found to be glycosylated [74].

#### 5.3.4 Other post-translational modifications

There are many other post-translational modifications (PTMs) and normally the discovery of these PTMs are discovered using high-throughput technologies such as MS-based proteomics. Phosphorylation for instance which is one of the most-studied PTMs remains little studied for AGR2. We, therefore, scoured the UniProt and PhosphoSite Plus, two of the most comprehensively curated protein databases for additional PTMs [96, 97]. In doing so, we found ten phosphorylation sites for AGR2 discovered from large proteomics datasets that have been deposited in PhosphoSite Plus but none from UniProt. These ten sites, including S36p, Y111p, T113p, T114p, S119p, Y124p, S146p, Y150p, Y152p and T157p were mostly derived from cancerous tissues and cell lines. Among these ten sites, only S119p, Y124p, Y150p and T157p have been published [98]. The published sites were discovered by a large-scale motif-targeting quantitative phosphoproteomic strategy using human lung adenocarcinoma cell line PC9. Whereas S146p was also independently reported by Mertins et al. from a proteogenomics study of breast cancer tissues [99]. Likewise, from PhosphoSite Plus, we found five deposited acetylation sites for K88, K89, K116, K165 and K166. Nevertheless, only K116Ac has been published by Wu et al. using non-small cell lung cancer A549 cell line sample to study the crosstalk between ubiquitylation and acetylation [100]. Indeed, K89 on AGR2 was also found to be ubiquitinated by an E3 ligase UBR5 [64]. It is noteworthy that one needs to exercise extra caution needs concerning PTMs identified from OMICS studies especially with regards to the false discovery rates (FDR) of these large-scale experiments. Besides, there is a current lack of sufficient validation, follow-up and functional characterisation, nonetheless, this information provides a starting point for further investigation. On top of that, PTMs of AGR2 that are outlined in this section have not been extensively studied and whether they have any roles concerning AGR2 secretion needs further justifications.

## 6 Clinical utility and functional impacts of iAGR2 and eAGR2 in cancer

Since AGR2 protein is highly overexpressed in tissues of several cancer types and can also be detected in the serum, plasma and urine, researchers have begun to investigate the prognostic value of AGR2. Tian et al. performed systematic review and meta-analysis of AGR2 expression in solid-tumour patients across 20 studies that include multiple cancer types in which the prognosis data are available [101]. Combined analyses showed that AGR2 overexpression leads to poor prognosis in all solid tumours. Notably, overexpression of AGR2 in breast cancer patients was significantly associated with poor overall survival compared to other cancer types. Further, AGR2 was also found in mass spectrometry (MS) analysis of prostate cancer tissues and AGR2 expression with LOX5 can be used as prognostic markers of biochemical recurrence [102]. This suggests that AGR2 can be used as a predictive marker for overall survival in solid tumour patients.

There are several studies aimed to detect the level of eAGR2 in the body fluids of cancer patients and determining the usefulness of eAGR2 for cancer diagnostics or prognostics. Studies describing the potential use of eAGR2 as a biomarker in cancer patients are summarized in Table 2. Wayner and colleagues developed an ELISA assay that can detect AGR2 protein in the voided urine of prostate cancer patients but not in the urine of normal samples, suggesting the potential use of eAGR2 as diagnostic marker in prostate cancer [103]. Moreover, using RT-PCR, AGR2 transcripts can be detected in the urine sediments of prostate cancer patients undergoing prostate biopsy [104]. The expression pattern of AGR2 is similar to the common prostate-specific antigen (PSA) marker, suggesting that AGR2/PSA expression ratio might be useful to discriminate prostate cancer patients from healthy individuals. The same research group also developed a highly sensitive targeted MS assay to quantify secreted AGR2 protein in human urine and serum. Using urine from prostate cancer patients, there was a significant difference between the urinary AGR2/PSA concentration ratios between non-cancerous and cancerous tissues, indicating the clinical utility of AGR2/PSA as a biomarker in prostate cancer [105]. However, it is important to note that the mere inclusion of AGR2 or PSA has no value in the discrimination of cancer and non-cancer samples. This is because AGR2 can also be detected in plasma and circulating tumour cells (CTCs) of prostate samples especially those with metastatic disease versus normal male controls [35]. However, plasma AGR2 levels do not correlate with PSA levels. Related to this, AGR2 could also be detected in the voided urine of bladder cancer patients but not in normal samples [106].

**Table 2:** Studies illustrating that eAGR2 can be detected in bodily fluids of cancer patients.

Cancer Type	Type of bodily fluids	Amount of detected AGR2	Method of detection	References
Prostate	Plasma	CSPC: Median (range) 105 (20–285) ng/ml CRPC: Median (range) 75 (10–2500) ng/ml NE-CRPC: Median (range) 945 (50–1,035) ng/ml	ELISA	[35]
		Range 1.26–181 pg/ml	ELISA	[103]
		Range 12.1–963.3 pg/100μg	Mass spectrometry	[105]
		Urine AGR2/PSA transcript ratio 0.68 (p = 0.015)	RT-PCR	[104]
	Serum	Serum tPSA = 0.53 (p = 0.66) Serum % fPSA = 0.59 (p = 0.22)	RT-PCR	
Ovarian	Plasma	OD450 = 0.15–1.5	ELISA	[107]
	Serum	-	ELISA	[108]
	Plasma	Range 2–18 ng/ml	ELISA	[39]
	Plasma	Median 765 pg/ml	ELISA	[119]
Bladder	Urine	OD450 (p = 0.012) AUC = 0.73	ELISA	[106]
Lung	Serum	Mean ± SD: 6.66 ± 6.18 ng/ml (p < 0.001)	ELISA	[120]
Nasopharynx	Serum	Median (range) NPC: 4.84 (0.12–15.26) ng/ml	ELISA	[121]
Breast	Serum	Median (range) 5.70 (2.79–13.73) ng/ml	ELISA	[121]
Pituitary	Serum	Mean ± SD 250.10 ± 79.14 ng/ml	ELISA	[122]
Pancreatic	Plasma Serum	AUC = 0.46 (p = 0.38) AUC = 0.58 (p = 0.00129)	ELISA	[123]
	Plasma	Mean = 8.8 μg/L Median = 2.1 μg/L	ELISA	[124]

AGR2 has also been detected in the plasma of ovarian cancer patients using ELISA. In this instance, AGR2 concentrations were found to be higher in stages II and III patients and were similarly elevated in patients with both serous and non-serous tumours [107]. Recently, incorporation of AGR2 in a panel of markers that includes CA125, HE4, CHI3L1, PEBP4 has been reported to improve the early detection of ovarian cancer up to 1 year before diagnosis [108]. This panel of markers demonstrated 85.7% sensitivity and 95.4% specificity and provided higher predictive values compared to using the classical serum cancer antigen CA125 alone.

## 7 Therapeutic strategies

The overexpression of AGR2 and its pro-oncogenic features indicate that AGR2 is a clinically-relevant anti-tumour target, hence researchers have begun developing antibodies against AGR2. For example, a specific aptamer against AGR2 was developed using the Systematic Evolution of Ligands by EXponential enrichment (SELEX). The aptamer C14B is 87-nucleotide long and can bind to AGR2 with high affinity but not to the controls thrombin, trypsin and BSA [109]. From this, an allosteric molecular beacon against AGR2 was further developed for AGR2 sensing using fluorescent flow cytometry analysis. Arumugam et. al. reported a murine blocking antibody against AGR2 that can reduce growth and metastasis of pancreatic cancers and suppress AGR2 client protein C4.4a, hence there are possibilities to use therapeutic agents to abrogate extracellular metastatic receptor activities [70]. Independently, one study developed a humanized antibody 18A4 that targets AGR2 [110]. Mouse xenograft study demonstrated that this antibody was able to inhibit xenograft tumour growth. A subsequent study showed that 18A4 is capable of detecting eAGR2, whereas the combination of both 18A4 and bevacizumab (targeting VEGF-A) was shown to inhibit tumour growth in an ovarian cancer xenograft model [75]. Qudsia et. al. utilized this 18A4 antibody sequence to generate a lentivirus based scFv optimization library that can be used to screen high-affinity variable domains and to improve binding affinity [111]. A pre-clinical in-vitro and in-vivo study showed that the same antibody was able to inhibit lung cancer growth. Treatment of lung cancer cell lines with monoclonal 18A4 antibody activated the p53 pathway, resulting in reduced cell proliferation [112]. Treating the mouse xenograft model with this monoclonal antibody led to impaired angiogenesis and reduced the tumour size without exerting adverse effects to major organs. This is the first study that showed such promising result of antibody-based therapy targeting AGR2, suggesting that this molecule is an important target in cancer development. Another rather exciting finding is the production of two humanized monoclonal anti-AGR2 antibodies, when incubated with human blood, resulting in cell lysis of prostate cancer cells containing eAGR2 [113].

## 8 Discussion and concluding remarks

**Figure 4:**
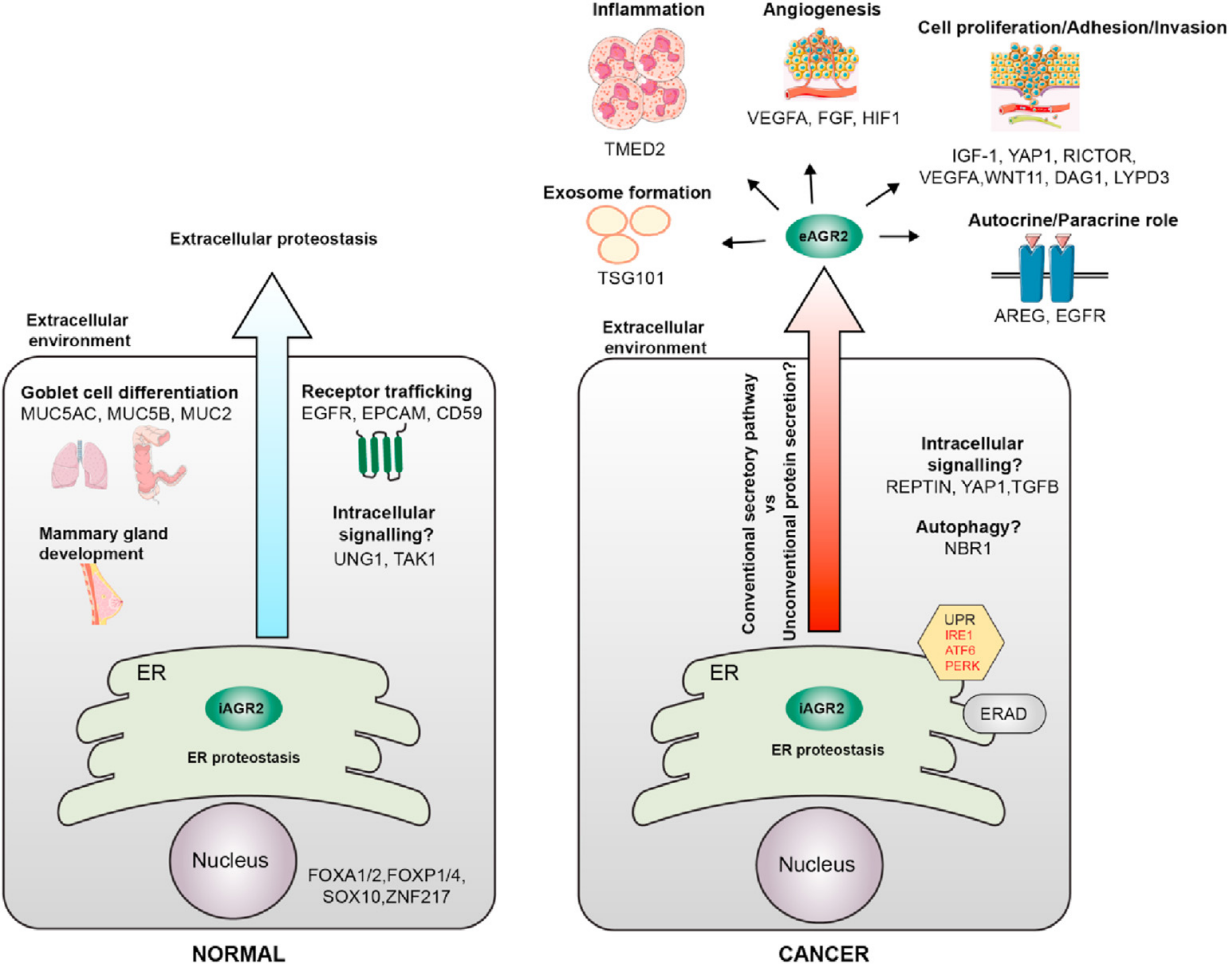
Emerging roles of iAGR2 and eAGR2 in normal and cancer conditions. AGR2 normally resides in the ER predominantly in its dimeric form and involves in the ER proteostasis through its protein disulphide isomerase activity. In a normal condition, AGR2 engages in the goblet differentiation that is responsible for secreting mucus in the respiratory and intestinal tracts that protects them from pathogenic infection. AGR2 is also essential for milk production during normal mammary gland development as well as receptor maturation and trafficking. In cancer cells, ER protein synthesis machinery is challenged with high mutant protein folding demands causing an ER stress that in turns activates UPR. In this context, AGR2 is actively regulated by the UPR pathways possibly via the IRE1α and ATF6 arms that can impact on AGR2 functions and export from the ER. There could also be an interplay of UPR/ERAD/Autophagy pathways. AGR2 can also escape the ER and be secreted into the extracellular environment in cancer. The presence of eAGR2 in the extracellular environment can contribute to the hallmarks of cancers such as enhancing cell proliferation, metastasis and dissemination, inflammation and angiogenesis. The mechanisms of AGR2 secretion are beginning to be elucidated. These include, among others, the association of its structure-function variants, possible PTMs and dimeric-monomeric regulations.

From the accumulated evidences presented in this review and others, it can be postulated that there is a dual role of AGR2 in promoting cancer development (Figure 4). First, there is an intracellular AGR2 whose function previously described as a catalyst in the ER proteostasis to cope with cancer cells secretory demands as well as controlling the secretome. Second, an extracellular AGR2 that involves in pro-oncogenic signalling in epithelial tumorigenesis, ECM remodelling, inflammation responses as well as angiogenesis. However, the mechanisms of how AGR2 can escape the ER is poorly understood. The functions of eAGR2 in the physiological and pathophysiological processes have garnered significant interest and attention. These growing knowledge on eAGR2 can contribute to the better insight and mechanistic models on how AGR2 modulates the cancer phenotype and progression. Gene editing such as the emerging CRISPR/Cas technology can be utilized to knockout AGR2 or to introduce AGR2 knock-in mutants in cell-based assays to understand the precise AGR2 roles and its mechanisms of secretion. Additionally, this secreted AGR2 can be found in the bodily fluid of cancer patients and the level of the expression can be distinguished from normal patients suggesting that AGR2 can be used as a cancer marker for diagnosis or prognosis.

The advent of OMICs platform has led to the rapid discovery of new biomarkers for human diseases. However, it is surprising that there are only a few markers that have been approved or have an impact on diagnosis/prognosis. Perhaps, the current tumour marker tests remained unchanged for decades. Based on the current data, AGR2 might be an appealing marker cancer diagnosis/prognosis. Diagnostics tool such as microfluidic detection device or biosensor can be developed to specifically and sensitively detect AGR2 for early cancer diagnosis using bodily fluids. Inclusion of AGR2 with other biomarkers may increase the sensitivity and specificity of detecting cancer and this has been demonstrated in the case of early detection of ovarian cancer [108]. Therapeutics strategies that aim to target AGR2 in cancer thus far has demonstrated promising results. However, more studies need to be persuaded to gain more insights into the efficacy and its pharmacogenomics utilities. Perhaps, targeting AGR2 and its oncogenic interactome might be needed to synergistically improve cancer treatment.

## Declarations

### Author contribution statement

All authors listed have significantly contributed to the development and the writing of this article.

### Funding statement

This work was supported by Dana Impak Perdana, 10.13039/501100004515Universiti Kebangsaan Malaysia (DIP-2018-011).

### Competing interest statement

The authors declare no conflict of interest.

### Additional information

No additional information is available for this paper.
